# Unilateral instrumented fixation for cervical dumbbell tumors

**DOI:** 10.1186/1749-799X-9-2

**Published:** 2014-01-20

**Authors:** Kei Ando, Shiro Imagama, Zenya Ito, Kazuyoshi Kobayashi, Junichi Ukai, Akio Muramoto, Ryuichi Shinjo, Tomohiro Matsumoto, Hiroaki Nakashima, Yoshihiro Nishida, Naoki Ishiguro

**Affiliations:** 1 Department of Orthopaedic Surgery, Nagoya University Graduate School of Medicine, 65-Tsurumai, Nagoya City, Aichi 466-8550, Japan

**Keywords:** Unilateral instrumented fixation, Cervical dumbbell tumors, Pedicle screw misplacement

## Abstract

**Purpose:**

The purpose of this study was to describe the radiological outcomes in patients with unilateral instrumented fixation for cervical dumbbell tumors.

**Patients and methods:**

Fourteen consecutive individuals were included in the present study. We included Eden type II and III tumors in this cohort study and analyzed fixed segment fusion rates, screw failure with multiplanar reconstruction computed tomography (CT) scan radiographs and lateral radiographs with flexion-extension dynamic views, and immediate postoperative and last follow-up radiographs after surgery.

**Results:**

The mean follow-up was 105.4 months. There were six men and eight women ranging in age from 32 to 70 years (mean age, 48 years). Twenty pedicle screws (PSs) and 11 lateral mass screws (LMSs) were used in total. There were seven patients with only PSs, four with only LMSs, and three with PSs at C2 and LMSs at C3. PS misplacement occurred in three screws of insertions including two screws with grade 1 misplacement and one screw with grade 2 misplacement, and no grade 3 misplacement occurred. All screws breached the lateral wall with no apparent superior or inferior misplacement. None of the LMSs were misplaced. Fortunately, no complication could be directly attributed to screw insertion. Radiological evidence showed that all patients achieved successful fusion with no screw loosening or breakage. However, two patients who received only LMS fixation had degenerative spondylolisthesis at the upper fusion segment at the last follow-up.

**Conclusions:**

Grade 2 PS misplacement occurred in one screw of insertions. Unilateral pedicle screw fixation for cervical dumbbell tumors is a useful surgical method that can successfully fuse vertebrae with good postoperative alignment.

## Introduction

Unilateral facetectomy has been used with cervical spinal and spinal cord tumors such as those at the pedicle and the posterior portion of the vertebra and cervical dumbbell tumors. In an experimental study, isolated, unilateral, cervical facetectomy resulted in an average 31.6% decrease in strength as compared to an intact motion segment [1]. Moreover, fusion may include more than three vertebrae when bilateral instrumented fixation is employed because it is often impossible to insert pedicle and lateral mass screws into the affected side due to pedicle and lamina scalloping such as what is seen with dumbbell tumors. The obvious advantage of placing instrumented fixation on only one side of the cervical spine is that less damage occurs to the non-affected lamina of the vertebrae situated rostrally or caudally to the affected vertebra, and we do not lose an additional motion segment. Therefore, to reduce the fusion levels involved and preserve the motion segment, we have performed unilateral instrumented fixation via a one-stage posterior approach for these tumors. To our knowledge, no peer-reviewed, published studies have quantifiably evaluated radiographic data from unilateral instrumented fixation of the cervical spine. The objective of this retrospective study was to systematically evaluate the radiographic data from patients who received unilateral instrumented fixation in a one-stage posterior approach.

## Materials and methods

The study was conducted after approval from the Human Ethics Committee of the hospital. Written informed study consents to participate in this study were obtained from the patients.

Between 1998 and 2010, 35 patients received surgery for resection of cervical spinal and spinal cord tumors. Patients were excluded if they had a malignant tumor, a dumbbell tumor at the atlanto-axial level which did not require a facetectomy or involved multiple levels affecting more than three facets, any Eden type I tumor that did not need a complete facetectomy on the affected side, or an Eden type IV tumor which is usually treated with a ventral approach. Among these patients, 14 consecutive individuals (6 men, 8 women) were included in the present study. We included Eden type II and III tumors in this cohort study. We inserted screws into the contralateral side (non-affected side) because we could not insert into the pedicle scalloped (affected side) by the tumor.

### Surgical strategy

When only one facetectomy was performed during tumor resection, the fusion segments were the same as the affected levels. We inserted pedicle screws (PSs) when the pedicle diameter was sufficient and preoperative magnetic resonance (MR) angiography indicated no obvious dominant vertebral artery on the non-affected side. If there was an obvious dominant vertebral artery on the non-affected side, we used lateral mass screws (LMSs) to avoid pedicle screw misplacement. When the tumor invaded two facet joints and we had to resect these joints to remove a paravertebral tumor, unilateral three-vertebra fixation was performed.

### Surgical techniques

Using 3D computed tomography (CT), we acquired all relevant information on the involvement of any artery, bone, or other peritumoral structures prior to surgery. During surgery, we monitored somatosensory evoked potentials (SSEPs) and motor evoked potentials (MEPs) with the patient in prone position under general anesthesia. The neck was maintained with a Mayfield head clamp, and the shoulder girdles were pulled caudally and immobilized with a tape. We then exposed both facet joints entirely to remove the tumor on the affected side and to fuse the contralateral side to the lateral margins of the facet joints using instrumentation in a subperiosteal fashion. The point of the PS was slightly lateral to the center of the articular mass and close to the inferior margin of the inferior articular process of the cranially adjacent vertebra [2]. The entry point of the LMS was 1 mm medial to the midpoint of the facet joint [3].

Using a CT-based navigation system (VectorVision Compact, BrainLAB, Heimstetten, Germany), we made pedicle and lateral mass screw holes. The pedicle or lateral masses were drilled prior to any decompression, if needed. Then, we performed an open-door laminoplasty opening at the affected side and exposed the dural sac. We resected the lateral mass, transverse process, and facet at the affected level using Kerrison’s punch and drilled as needed to enlarge the longitudinal exposure. The dura was opened for an intradural tumor excision (Eden type II) using an operating microscope extended laterally over the nerve root sleeve. After removing this component of the tumor and sacrificing the entire affected spinal nerve root to prevent traction on the spinal cord during excision of a paravertebral tumor, we closed the dura in a watertight fashion and placed fat harvested from the subcutaneous tissue using fibrin glue. Then, the extraforaminal component with its distal stump and an encapsulated smooth surface tumor at the back side were exposed and carefully resected. Finally, the motion segments to be fused had their facet joints decorticated to insert the screws under fluoroscopic guidance.

Great care was taken to protect the facet joints above and below the instrumented levels. We determined the screw length predominantly through assessment of preoperative imaging. For PSs, the pedicle probe was inserted from the hole made under the CT-based navigation system before tumor resection to the lateral edge of the lateral mass, with the aimed transverse angle around 35°. The screw trajectory angle was only 30° to 35° from the sagittal plane because a larger inclination required additional surgical exposure [4]. As an exception, a more reduced angle was adopted for C2 because of its anatomic difference. Bicortical fixation was specifically performed for LMSs. We used a trajectory modified from the standard trajectories for screw placement [3,5]. We angulated the LMSs 20° to 25° laterally and superiorly to attain the best purchase of the lateral mass with minimal risk of neural or vascular injury; this is a modification of the Anderson technique [6]. We applied a rod or plate to prevent deformities that can occur with instability, followed by a bone graft using the spinous process and lamina resected to the decorticated lamina and facet joints of the non-affected side.

Finally, we performed a meticulous closure of the wound in layers over a drain. Postoperatively, patients were placed on bed rest for 3 to 4 days and then mobilized by the physiotherapist after removal of the drain. Postoperatively, they wore a cervical brace for 3 months.

### Radiographic data

**Figure 1:**
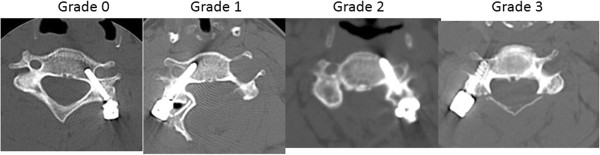
**Medial and lateral deviations of the screw classified according to the modified classification.***Grade 0*, no deviation and the screw was contained in the pedicle. *Grade 1*, deviation less than 1 mm (i.e., less than half of the screw diameter). *Grade 2*, deviation more than 1 mm and less than 2 mm (i.e., less than half of the screw diameter). *Grade 3*, deviation more than 2 mm and the direction of misplacement was medial, lateral, superior, or inferior. Grades 2 and 3 were considered critical deviations.

Two experienced examiners (Z.I. and A.M.) evaluated the preoperative, immediate postoperative, and last follow-up radiographs, CT scans, and MRI scans of the cervical spine. They assessed fusion rates, screw failure with multiplanar reconstruction CT scan radiographs and lateral radiographs with flexion-extension dynamic views, and general complications. Radiographs were all taken in a sitting position. The PS positions were routinely evaluated with CT (2-mm slices) before hospital discharge. The examiners considered two independent factors, the degree and the direction of misplacement, when evaluating cervical PS misplacement. Using CT axial scans, the whole length of each screw was obtained, and the medial and lateral deviations of the screw were classified into four grades according to the modified classification of Neo et al. [7]: grade 0, no deviation and the screw was contained in the pedicle; grade 1, deviation less than 1 mm (i.e., less than half of the screw diameter); grade 2, deviation more than 1 mm and less than 2 mm (i.e., less than half of the screw diameter: grade 3, deviation more than 2 mm and the direction of the misplacement was medial, lateral, superior, or inferior (Figure 1). Grades 2 and 3 were considered critical deviations.

## Results

### Clinical data

The mean follow-up was 105.4 months, with a range of 24 to 180 months (Table 1). There were six men and eight women ranging in age from 32 to 70 years (mean age, 48 years). Twelve (85.7%) patients had long tract signs with gait disturbance, seven (50.0%) patients had radicular symptoms, and three (21.4%) patients had bowel and bladder dysfunction. According to the Eden classification [8] of cervical spinal cord tumors, 11 patients had type II and 3 patients had type III tumors. The mean operative time was 322.4 min (range, 200–452 min), and the mean estimated blood loss was 172.3 ml (range, 40–600 ml).

**Table 1:** Clinical data

**Patient number**	**Age/sex**	**Affected levels**	**EBL (ml)**	**Operation time (min)**	**Clinical presentation**	**Follow-up (months)**	**Pathology**	**Type (Eden’s classification)**	**CT scalloping**
1	52/female	C4 to C5	50	250	GD, RS	180	Schwannoma	II	+
2	70/male	C6 to C7	40	240	GD	167	Schwannoma	II	+
3	56/female	C5 to C6	80	290	GD	165	Schwannoma	III	+
4	41/female	C2 to C3	55	310	GD	144	Schwannoma	II	+
5	38/female	C6 to C7	330	360	GD, BBD	130	Schwannoma	III	+
6	32/female	C6 to T1	90	300	GD, RS	134	Schwannoma	II	+
7	60/male	C3 to C4	60	310	GD, RS	119	Schwannoma	II	+
8	44/female	C2 to C3	449	450	GD, BBD	90	Schwannoma	III	+
9	39/male	C5 to C7	300	429	GD, RS	60	Schwannoma	II	+
10	39/male	C2 to C3	600	452	GD	47	Schwannoma	II	+
11	54/male	C4 to C5	98	352	RS, GD, BBD	46	Schwannoma	II	+
12	38/female	C5 to C6	100	285	RS	24	Schwannoma	II	+
13	47/male	C6 to T1	100	285	GD, RS	146	Schwannoma	II	+
14	55/female	C5 to C6	60	200	RS	24	Schwannoma	II	+

*BBD* bowel and bladder dysfunction, *GD* gait disturbance, *RS* radicular symptom.

### Radiographic evaluation

Twenty PSs and 11 LMSs were used in total (Table 2). There were seven patients (50%) with only pedicle screws, four (28.6%) with only lateral mass screws, and three (21.4%) with PSs at C2 and LMSs at C3. The implants used were a Ti-mini-VSP system (DePuy Spine Inc., Raynham, MA, USA) in six patients, the Vertex System (Medtronic Sofamor-Danek, Memphis, TN, USA) in four patients, and the OASYS System (Stryker Spine, Allendale, NJ, USA) in four patients. All screws had a diameter of 3.5 mm. The screw lengths were 14 to 18 mm in LMSs, 22 to 24 mm in PSs, and 20 mm in C2 PSs. PS misplacement occurred in three screws of insertions including two screws with grade 1 misplacement and one screw with grade 2 misplacement, and no grade 3 misplacement occurred. All screws breached the lateral wall with no apparent superior or inferior misplacement. None of the LMSs were misplaced. Fortunately, no complication could be directly attributed to screw insertion, and the patients experienced no postoperative neurological deterioration. Radicular symptoms resolved in all patients, gait disturbance due to myelopathy improved in all patients to some extent, and one patient had postoperative subcutaneous liquorrhea, which was absorbed conservatively.

**Table 2:** Radiographic description

**Patient number**	**Fusion area**	**Fusion methods**	**Screw length (mm)**	**Rod or plate**	**Misplacement (grade)**	**Malposition**	**Radiographic change**
1	C4 to C5	C4, C5: LMS	18, 18	Plate	0	-	
2	C6 to C7	C6, C7: PS	24, 24	Plate	0	-	
3	C5 to C6	C5, C6: LMS	18, 18	Plate	0	-	
4	C2 to C3	C2: PS, C3: LMS	20, 16	Plate	0	-	
5	C6 to C7	C6, C7: PS	24, 24	Plate	0	-	
6	C6 to T1	C6, C7, T1: PS	22, 24, 24	Rod	0	-	
7	C3 to C4	C3, C4: PS	22, 22	Rod	C5 grade 2	Lateral	
8	C2 to C3	C2: PS, C3: LMS	20, 14	Rod	C2 grade 1	Lateral	
9	C5 to C7	C5, C6, C7: PS	22, 22, 22	Rod	0	-	C4/C5 disc height narrowing
10	C2 to C3	C2: PS, C3: LMS	20, 16	Rod	0	-	
11	C4 to C5	C4, C5: LMS	14, 14	Rod	0	-	C4 spondylolisthesis
12	C5 to C6	C5, C6: LMS	24, 24	Rod	0	-	C5 spondylolisthesis
13	C6 to T1	C6, C7, T1: PS	22, 22, 22	Plate	C6 grade 1	Lateral	
14	C5 to C6	C5, C6: PS	24, 24	Rod	0	-	

*LMS* lateral mass screw, *PS* pedicle screw.

**Figure 2:**
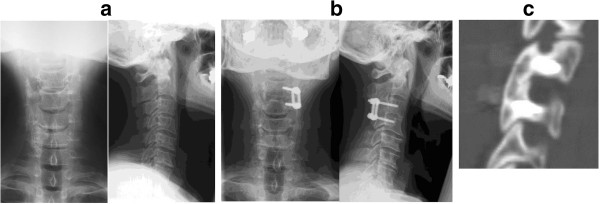
**Unilateral pedicle screw fixation (patient number 7). (a)** Plain radiography of the cervical spine of a 60-year-old man showed a positive pedicle sign at the left C4. **(b)** Plain radiography at the last follow-up showed a good union with no implant failure at the last follow-up. **(c)** Sagittal CT at the last follow-up.

**Figure 3:**
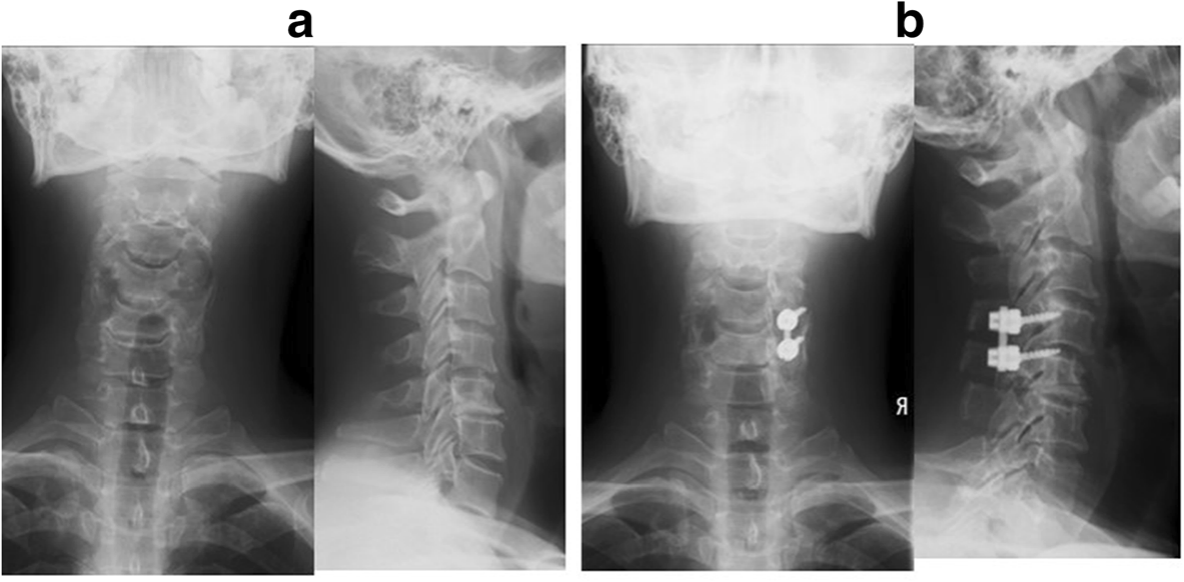
**Unilateral lateral mass screw fixation (patient number 11). (a)** Plain radiography of the cervical spine of a 54-year-old man showed a positive pedicle sign at the left C5. **(b)** Plain radiography showed C4 spondylolisthesis (2-mm increase in vertebral slip) which was evident at the last follow-up.

Radiological evidence showed that all patients achieved successful fusion without implant breakage at the last follow-up. Patients with only PSs (case numbers 2, 5, 6, 7, 9, 13, 14) or both PSs and LMSs (case numbers 4, 8, 10) had stable fixed segments in lateral flexion-extension radiographic views and achieved bone union on sagittal CT from the preoperative period to the last follow-up (Figure 2). However, two patients (50%: case numbers 11, 12) who received only LMS fixation had degenerative spondylolisthesis at the upper fusion segment (Figure 3). Fortunately they had neither instability with flexion-extension nor lucencies surrounding any screws at the last follow-up. One patient (case number 9) had narrowing of the disc height at the rostral area of the fused segment.

## Discussion

In spinal surgery with instrumentation, achieving fusion is very important. Some reports on lumbar unilateral instrumentation had fusion success rates of 91.9% to 100% of patients, and biomechanics studies demonstrated that unilateral fixation was sufficient for maintaining spine stability [9-12]. However, only a few biomechanics studies indicated clinical outcome [1,13-15]. Those reports indicated that cervical facetectomy resulted in a 29% to 31.6% decrease in strength as compared with an intact motion segment [1,13]. One study used a hydraulic torsion test to apply increasing amounts of torque to the subaxial spine until a unilateral facet dislocation occurred [15]. Conversely, indications are that there is little resistance to torque at a unilateral facetectomy site and instrumented fixation is needed.

The advantage of unilateral instrumented fixation is its use in fusing short segments of vertebrae. More than three vertebrae (extending to an additional rostral or caudal unaffected spinal level) must be fused when bilateral instrumented fixation is employed since we cannot insert pedicle and lateral mass screws into the affected side of a vertebra due to pedicle and lamina scalloping such as with dumbbell tumors. The downside of such a strategy is that the mobility of the additional motion segment is lost.

PS fixation is superior to other techniques in terms of promoting mechanical strength [16,17]. In this study, all cases with PSs alone (seven patients) and those with both PSs and LMSs (three patients) achieved fusion without malalignment. Although PS fixation has been criticized for the potential risk of serious injury to neurovascular structures such as the spinal cord, nerve root, and vertebral artery [7,18,19], it has great internal stability and the rate of pseudarthrosis is low [20]. Because even a grade 2 misplacement of a PS may be life-threatening, and because the navigation system is helpful in reducing the rate of complications and clinical symptoms connected to cervical PS misplacement, using a navigation system remains the safest approach [20].

We drilled the pedicle and lateral mass screw holes with guidance from the navigation system but inserted the screws using fluoroscopy after removing the tumors to decrease the chance of injury to the vulnerable spinal cord during techniques such as screw tapping. We inserted PSs when there was a sufficient pedicle diameter and the dominant vertebral artery was not obvious at the non-affected side on preoperative MR angiography. If a dominant vertebral artery was obvious at the non-affected side, LMSs were inserted to prevent complications from possible pedicle screw misplacement. Four patients received LMSs. Unilateral LMS fixation that had spondylolisthesis in 50% was not enough to preserve the postoperative alignment.

A human cadaveric study demonstrated that unilateral lateral mass fixation was less stable than an intact specimen averaged over all ranges of motion [14]. In this report, the unilateral C5 to C6 lateral mass construct was associated with an increased C5 to C6 range of motion (110.1% of normal), although the bilateral C5 to C6 lateral mass construct reduced the range of C5 to C6 motion to 33.6% of normal. We considered that mechanical failure occurred because unilateral lateral mass screw fixation is not enough primary stabilization and cannot preserve the postoperative alignment until bone union was required. If LMSs must be used in a unilateral fixation due to an insufficient pedicle diameter or an obvious dominant vertebral artery at the non-affected side, prolonged rigid external fixation such as the use of an Adfit brace may be needed until union is achieved.

This study has several limitations. First, treating different cervical spinal and spinal cord tumors in this study may have influenced outcomes. Furthermore, given that we had very specific inclusion criteria, we could gather only a small size sample for this study and there was no comparative group such as no fixation or bilateral fixation. An anterolateral approach is also useful to remove the tumor, especially for Eden type III [21]. Nevertheless, we will continue to pursue the prospective case cohort study. We believe that the design will improve as cases accumulate.

## Conclusions

Unilateral pedicle screw fixation for cervical dumbbell tumors is a useful surgical method that can successfully fuse vertebrae with good postoperative alignment. Prolonged rigid external fixation such as the use of an Adfit brace for unilateral lateral mass fixation may be needed until union is achieved.

## Competing interests

The authors declare that they have no competing interests.

## Authors’ contributions

AM and TM have made substantial contributions to the conception, design, and interpretation of data. SI has made substantial contributions to the conception, design, analysis, and interpretation of data. He has also been involved in drafting the manuscript. KA, ZI, KK, JU, RS, HN, YN, and NI have made substantial contributions to the conception, design, and acquisition of data. All authors have been involved in revising the manuscript critically for important intellectual content and have read and approved the final manuscript.
